# Artificial Intelligence–Driven Graph Methods for Microbiome Research: A Systematic Review

**DOI:** 10.1002/wsbm.70016

**Published:** 2026-10-29

**Authors:** Cedrique Shum‐Tim, Paromita Islam, Jacqueline L. Boyajian, Ahmed Abosalha, Amal Kassab, Madison Santos, Kinaan Aamir Khan, Kiri Stern, Amin Emad, Satya Prakash

**Affiliations:** ^1^ Biomedical Technology and Cell Therapy Research Laboratory, Department of Biomedical Engineering, Faculty of Medicine and Health Sciences McGill University Quebec Canada; ^2^ Computational Biology and Artificial Intelligence Laboratory, Department of Electrical and Computer Engineering, Faculty of Engineering McGill University Quebec Canada; ^3^ Mila – Québec AI Institute McGill University Quebec Canada; ^4^ Pharmaceutical Technology Department, Faculty of Pharmacy Tanta University Tanta Egypt; ^5^ Department of Quantitative Life Sciences, Faculty of Interfaculty Studies McGill University Quebec Canada

**Keywords:** deep learning, graph representation learning, high‐throughput sequencing, metagenomics, microbiome, network science, networks

## Abstract

The complexity of high‐throughput sequencing data in microbiome research necessitates advanced analytical methods to decipher microbial community structures and interactions. Graph‐based methods, which model taxa as nodes and their relationships as edges, have emerged as a powerful framework for this purpose. However, the rapid evolution of these techniques, from classical network analysis to modern graph representation learning, has created a fragmented and complex methodological landscape. This systematic review provides a comprehensive synthesis and structured overview of the field. We systematically review the literature and propose a three‐part classification to categorize the primary contribution of each study: (1) novel methods for microbial network representation, (2) applications of classical graph analysis, and (3) the emerging use of graph representation learning for predictive modeling. We summarize the most frequent applications within each class, including keystone species identification, community clustering, and graph‐based disease classification. Furthermore, we identify critical cross‐cutting challenges, such as the lack of standardized tools and the need for improved model interpretability. By structuring the current landscape of graph‐based methods, this review serves as a crucial guide for researchers to select appropriate models and highlights key gaps to direct future research.

## Graphical abstract

Multi‐omics and microbiome‐derived datasets were converted into deterministic and probabilistic network representations for downstream classical graph analysis and graph representation learning. This framework enables link prediction, relationship discovery, and node clustering to improve interpretation of complex biological interactions.

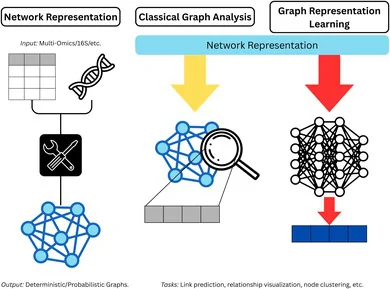

## 1 Introduction

The microbiome, the intricate community of microorganisms inhabiting diverse ecological niches, plays a fundamental role in host health and environmental balance. This community comprising bacteria, archaea, fungi, and viruses is integral to processes such as nutrient metabolism, immune system development, and protection against pathogens. A growing body of evidence links imbalances in the microbiome, or dysbiosis, to a wide range of conditions, including obesity (Kincaid et al. 2020), diabetes (Qin et al. 2012), and allergies (Vatanen et al. 2016). To study these communities, researchers typically use high‐throughput sequencing techniques like 16S rRNA gene sequencing or shotgun metagenomics. These methods generate vast amounts of data, often processed using tools like QIIME (Bolyen et al. 2019; Caporaso et al. 2010) into feature tables of microbial taxa. However, this data is inherently high‐dimensional, sparse, and compositional, making it difficult to decipher the complex web of microbial interactions using traditional statistical methods alone.

To address this challenge, network science has emerged as a powerful framework for modeling the intricate relationships within microbial ecosystems. By representing microbial taxa as nodes and their associations (such as correlation or co‐occurrence) as edges, researchers can construct networks that visualize and quantify community structure. The initial step of translating raw microbial data into a meaningful graph is itself a critical and active area of research, with various methods proposed to create robust microbial network representations.

Once a network is constructed, it can be analyzed to yield significant biological insights. For decades, researchers have applied classical graph analysis methods to these networks to identify keystone species through centrality measures (Peschel et al. 2021), discover functional modules via community detection (Deng et al. 2022), and explore ecological dynamics (Melnyk et al. 2023). More recently, the field has advanced toward adopting sophisticated graph representation learning (GRL) techniques. Methods like geometric deep learning can automatically learn rich, low‐dimensional feature representations of nodes and entire graphs, moving beyond descriptive analysis to powerful predictive modeling (Gilmer et al. 2020). This evolution enables researchers to classify communities, predict host–microbe interactions, and identify novel biomarkers with greater accuracy (Bucci et al. 2016). For example, graph‐embedding and graph‐convolutional approaches such as MOGONET (Wang, Shao, et al. 2021) have been used to identify candidate microbial biomarkers directly from community data while integrating multiple omics layers.

Despite the growing adoption of these graph‐based methods, the field is expanding rapidly and becoming fragmented. A wide array of techniques, from network inference algorithms to state‐of‐the‐art graph neural networks, are being applied, often without a clear consensus on best practices. Open questions include the choice of network‐inference method (e.g., correlation‐based versus conditional‐dependence approaches) (Weiss et al. 2016), the handling of compositionality and data normalization (Gloor et al. 2017), the criteria for edge thresholding and network sparsification (Röttjers and Faust 2018), and the strategies for validation and benchmarking (Faust 2021). For researchers new to the field, navigating this diverse and complex methodological landscape is a significant hurdle. To our knowledge, no systematic review has comprehensively cataloged and synthesized the literature that spans this entire workflow, from initial network construction to classical analysis and modern GRL for analysis.

Therefore, this systematic literature review aims to provide a structured and comprehensive overview of the current state of graph‐based methods in microbiome research. We identify, categorize, and synthesize the existing literature into three categories: (1) methods for microbial “network representation”, (2) “classical graph analysis” techniques on microbial networks, and (3) emerging “graph representation learning” methods. By classifying studies into these three core areas, we analyze trends in data usage, tool availability, and key methodological challenges, ultimately providing a critical synthesis of the field and a roadmap for future research. We focus on bacteria, archaea, fungi, and viruses because these dominate the graph‐based microbiome studies reviewed here; protozoa and other microbial eukaryotes were not a specific focus of the retrieved literature, though the graph‐based methods described are in principle agnostic to the taxonomic identity of the nodes.

Prior reviews from the ML4Microbiome initiative have surveyed statistical and machine learning techniques in microbiome research and cataloged the broader software ecosystem, including challenges of standardization, explainability, and reproducibility (Moreno‐Indias et al. 2021; Marcos‐Zambrano et al. 2023; Papoutsoglou et al. 2023). Our review is complementary but distinct in scope: rather than surveying machine learning broadly, we focus specifically on graph‐based methods and organize the literature along the full graph‐analysis workflow, from network representation through classical graph analysis to graph representation learning.

## 2 Article Selection

To conduct this systematic literature review, a strict and reproducible search strategy was employed to identify relevant articles and studies pertaining to the integration of graph‐based analysis methods in microbiome research. Three different bibliometric databases (PubMed, IEEE, and Google Scholar) were searched for articles with the following keywords: (“graph representation” OR “network science” OR “graph learning”) AND “microbiome”. For PubMed and IEEE, the query was done over the titles and abstracts, with all search results being considered for the study. For Google Scholar results, the 200 most relevant studies pertaining to the search query were collected. This resulted in a total of 241 records from all three databases initially considered. Once the initial collection was achieved, the titles and abstracts were manually scanned to exclude articles deemed irrelevant to the scope of this review. This screening step reduced the collection to 185 studies. Subsequently, duplicate entries and review articles were identified and removed, resulting in a refined set of 144 studies. Finally, 7 articles (4.9% of the 144 eligible studies) that were inaccessible due to paywalls or unavailability were excluded, leaving a final dataset of 137 studies for detailed analysis (see Figure 1). We acknowledge that excluding inaccessible articles may introduce a degree of selection bias; however, given the small proportion affected, we do not expect it to materially alter the methodological trends reported here. This systematic review followed the general PRISMA framework for study identification and selection (Figure 1) but is a methodological review and therefore does not implement all PRISMA items designed for quantitative clinical syntheses.

**FIGURE 1:**
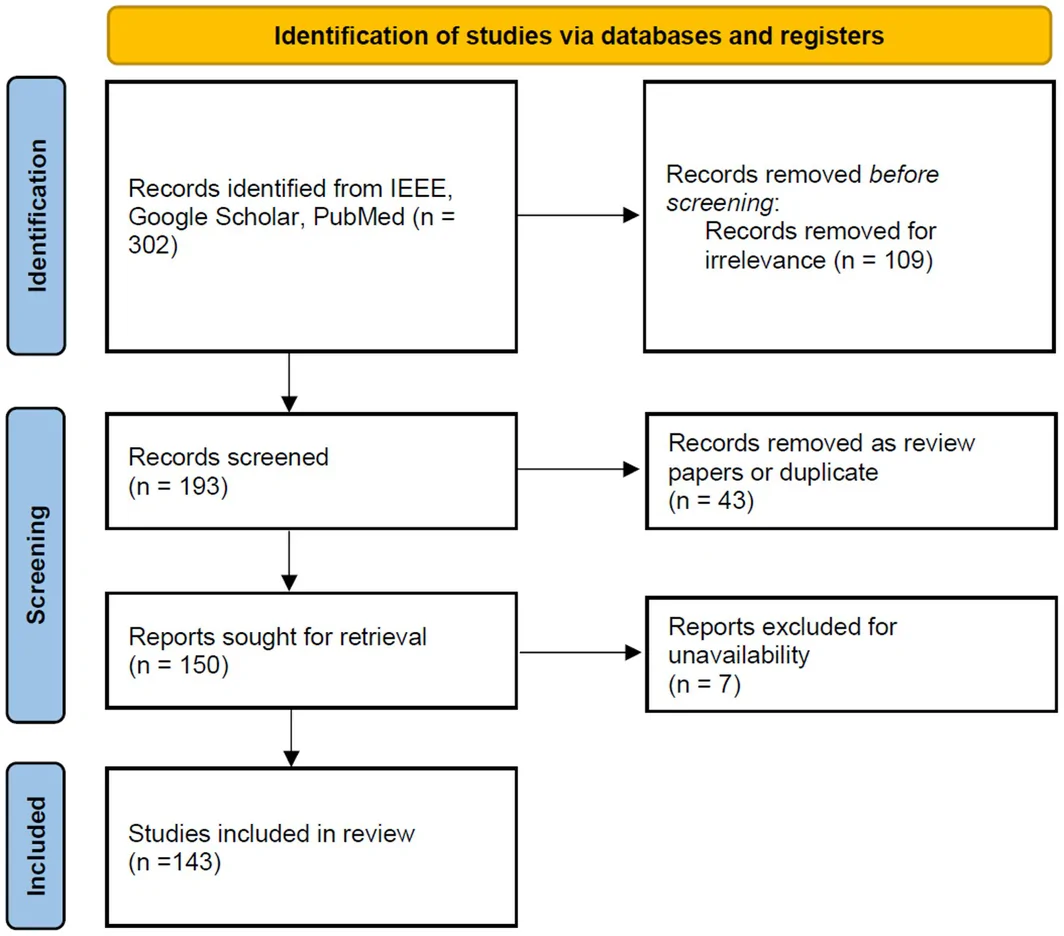
Flowchart of the literature search and study inclusion process. The diagram summarizes the identification of articles via database searches, the screening for relevance, the full‐text eligibility assessment, and the final inclusion of studies that met the criteria for this systematic review on graph‐based methods in microbiome research.

A few limitations of this search strategy should be noted. The use of three databases and a focused set of graph‐related keywords, combined with restricting Google Scholar to the 200 most relevant results, may have excluded relevant studies that use different terminology (e.g., “co‐occurrence network” or “microbial association network” without the graph‐specific terms) or that are indexed elsewhere. Our intent was to capture studies explicitly framed around graph and network methodology rather than to enumerate every microbiome study that incidentally constructs a network.

Consistent with the methodological focus of this review, we did not perform a formal risk‐of‐bias or study‐quality assessment of the type used in clinical systematic reviews, as the included studies are largely methodological contributions rather than comparative clinical investigations. Instead, where possible we appraised each method along dimensions relevant to computational reproducibility, namely code and data availability, implementation language, and software maintenance, which are summarized in the Availability of Tools section and Tables [Link], [Link].

## 3 Study Classification

To systematically categorize and analyze the reviewed articles, we classified them into three distinct categories based on their primary focus and methodological approach. This classification ensures clarity in understanding the contributions of each study and facilitates comparative analysis.

Each study was assigned to a single category according to its primary methodological contribution. Studies that span multiple areas were classified by their principal novel contribution: a study whose main contribution is a new network‐construction technique was placed in the network‐representation category even if it subsequently applied classical analysis, whereas a study whose main contribution is a learned low‐dimensional representation was placed in the graph‐representation‐learning category. Where a method is illustrative of concepts in more than one section, it may be discussed in multiple places but is counted only once in the category totals (network representation, *n* = 80; classical graph analysis, *n* = 8; graph representation learning, *n* = 49).

### 3.1 Methods for Microbial Network Representation

This category includes articles that primarily focus on developing new or innovative methods for constructing network representations from microbial data (*n* = 80, Table S1). These studies introduce novel techniques for computing microbial interactions, alternative node definitions, or the integration of different data to form more comprehensive microbial networks. The emphasis in these studies is on the methodology of network formation and visualization rather than subsequent analysis (Peschel et al. 2021; Bucci et al. 2016; Schwager et al. 2017; Vinciotti et al. 2022, 2024; Fang et al. 2015; Shen and Solis‐Lemus 2020; Ha et al. 2020; Liu et al. 2022; Price et al. 2009, 2010; Tian et al. 2020; Jiang et al. 2020, 2015; Feng, Peng, et al. 2022; Balaji et al. 2022; Zhao et al. 2021; Cougoul et al. 2019; Gibson and Gerber 2018; Gibson et al. 2021; Ma 2021; May et al. 2016; Zoppi et al. 2021; Liu, Pan, et al. 2021; Reiman et al. 2021; Hu et al. 2022, 2023; Tavakoli and Yooseph 2019; Lo and Marculescu 2017; Yooseph and Tavakoli 2022; Lima‐Mendez et al. 2015; Kim et al. 2019; Lugo‐Martinez et al. 2019; Gadhia et al. 2023; Steinway et al. 2015; Laccourreye et al. 2022; Zhang and Deng 2021; Sedlar et al. 2016; Gerber et al., n.d.; Sazal et al. 2021, 2020; Imangaliyev et al. 2015; Reiter et al. 2022; Baranwal et al. 2021; McGeachie et al. 2016; Hannigan et al. 2018; Prost et al. 2021; Greenblum et al. 2012; Ganesan et al. 2020, 2017; Mamet et al. 2019; Paropkari et al. 2016; Faust et al. 2012; Martin et al. 2007; Noyes et al. 2018; Hintikka et al. 2023; Ma and Li 2019; Estrada‐Peña et al. 2018; Loftus et al. 2021; Xu et al. 2023; Labus et al. 2019; Ma, Wang, et al. 2020; Vandeputte et al. 2017; Galli and Fasanelli 2020; Larsen and Claassen 2018; Richards et al. 2014; van den Bergh et al. 2012; Mac Aogain et al. 2021; Kuntal et al. 2019; Champion et al. 2024; Ciucci et al. 2017; Bernstein et al. 2019; Ban et al. 2015; Zhang et al. 2015; Soriano et al. 2023; Narayana et al. 2021; Friedman and Alm 2012; Kurtz et al. 2015; Yoon et al. 2019; Long and Luo 2019).

### 3.2 Classical Graph Analysis Methods on Microbial Networks

Articles in this category employ well‐established graph analysis techniques to examine microbial networks (*n* = 8, Table S2). These studies leverage classical graph‐theoretic approaches to extract meaningful insights from microbial data. The primary contribution of these studies lies in their application of graph analysis methods rather than proposing new ways to construct microbial networks (Zou et al. 2017; Chen et al. 2017; Lymberopoulos et al. 2022; Lutz et al. 2025; Ling et al. 2016; Copeland et al. 2015; Vernocchi et al. 2020; Hall et al. 2019).

### 3.3 Graph Representation Learning on Microbial Networks

This category encompasses studies that explore or introduce new architectures of GRL for microbial network analysis (*n* = 49, Table S3). These articles propose novel methods for learning and/or analyzing low‐dimensional network representations. The primary focus here is on enhancing predictive performance, feature extraction, and knowledge discovery through improved representation learning methods as opposed to constructing microbial networks or using non‐embedding‐based network analysis techniques (Deng et al. 2022; Melnyk et al. 2023, 2020; Hajiramezanali et al. 2020; Xu et al. 2022; Lu et al. 2023; Gautam et al. 2022; Zeng et al. 2022; Feng, Wu, et al. 2022; Cheng et al. 2022; Long, Wu, Liu, et al. 2020; Ito et al. 2021; Jiang et al. 2021, 2022; Long, Wu, Kwoh, et al. 2020; Gong et al. 2022; Liao et al. 2023; Zhu et al. 2020, 2023; Shtossel et al. 2023; Cai et al. 2010; Tan et al. 2022; Ma et al. 2023; Lei and Wang 2020; Wang et al. 2017; Chen and Lei 2022; Ciucci et al. 2020; Shi et al. 2023, 2022; Pi et al. 2022; Liu, Liu, et al. 2021; Ma, Zhao, and Ma 2020; Liu et al. 2024, 2018; Yang et al. 2022; Yousefi et al. 2023; Wang, Shao, et al. 2021; Hasanzadeh et al. 2022; Pust and Tümmler 2022; Fernandez et al. 2015; Khan and Kelly 2019; Yin et al. 2020; Pan et al. 2023, 2022, 2024; Xue et al. 2022; Tian et al. 2023; Burkholz et al. 2021; Wang, Lei, and Pan 2021).

## 4 Results

All of the methods gathered for the corpus of this review, regardless of their goal, use some form of network representation of microbial data (see Figure 2 for a flowchart illustration of the typical steps involved in conducting a microbial network analysis). Among these, *n* = 80 were in Category 1 (network representation), *n* = 8 were in Category 2 (classical graph analysis), and *n* = 49 were in Category 3 (graph representation learning), which we will discuss in detail below. However, before delving into the graph‐based methods used in microbiome research, we first outline the most important and recurring types of data studied in this review.

**FIGURE 2:**
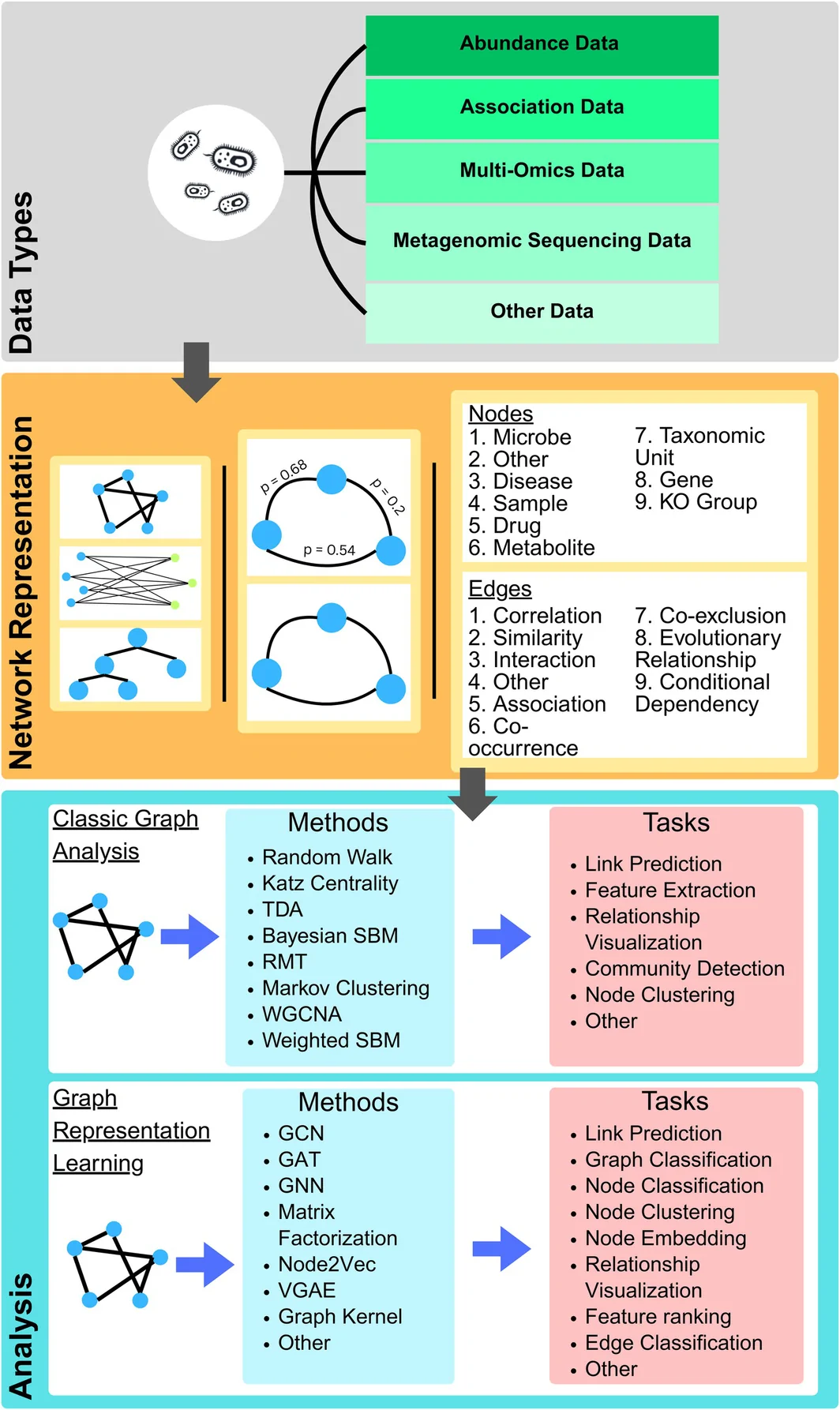
Flow chart illustrating the typical steps and processes involved in conducting microbial network analysis, as observed in the reviewed literature.

### 4.1 Microbial Data Types

Graph‐based approaches have been applied across a range of data types, reflecting the complexity of microbiome ecosystems and their interactions with host environments. While this section does not aim to cover every possible data type, it focuses on those that are most relevant to the methods that are being reviewed. Each type presents unique challenges and opportunities for graph‐based modeling, from capturing intricate ecological relationships within microbial communities to integrating multi‐omics data for a holistic understanding of microbiome function and can be used in conjunction with each other to gain more insights. Recognizing and addressing the distinct characteristics and limitations of these data types is critical to designing effective graph‐based methods and unlocking deeper biological insights. Note that different data types could be used and combined with other data types within a single study.

#### 4.1.1 Microbial Abundance Data

Microbial abundance data was the most prevalent data type used in the studies we reviewed, appearing in 60.58% (*n* = 83) of all the included articles. This data represents the composition of microbial communities in various environments, such as the human gut, soil, or water. It can be measured as absolute abundance, indicating actual microbial counts, or relative abundance, representing proportions of taxa within a community. Typically, this data is organized into operational taxonomic unit (OTU) or amplicon sequence variant (ASV) tables, where rows correspond to microbial taxa and columns to individual samples (Knight et al. 2018). These tables are often generated through either shotgun metagenomic sequencing or 16S rRNA gene sequencing, which provide snapshots of microbial communities at varying levels of taxonomic resolution. Shotgun sequencing offers the advantage of broader functional and taxonomic insights, while 16S rRNA sequencing is cost‐effective and widely used for taxonomic profiling. Regardless of the method, microbial abundance data forms the foundation for many graph‐based analyses, where taxa are represented as nodes and their relationships are captured as edges.

#### 4.1.2 Association Data

Another commonly used data type in the articles we reviewed was association data (*n* = 24), equivalent to 17.52% of the articles. In microbiome research, association data typically refers to known and quantified relationships between given variables (microbe, drug, disease, metabolome, etc.) that were previously computed using various statistical methods and made public. For example, the Human Microbe‐Disease Association Database (HMDAD) is a curated database that provides comprehensive information on the associations between human diseases and the human microbiome (Ma et al. 2017). One should note, however, that association data inherently represents correlations rather than causation, necessitating further experimental or computational validation to infer causal relationships.

#### 4.1.3 Multi‐Omics Data

Multi‐omics data was used in 15.33% (*n* = 21) of the included articles. Multi‐omics data in microbiome research refers to the integration and analysis of multiple layers of biological data obtained from the same microbiome samples, capturing diverse molecular features and processes. These layers typically include genomics (DNA), transcriptomics (RNA expression), proteomics (proteins), metabolomics (metabolites), and epigenomics (epigenetic modifications) (Heintz‐Buschart and Westerhuis 2022). By combining these datasets, researchers gain a holistic understanding of the microbiome's composition, functional capabilities, and interactions with its environment or host. Multi‐omics approaches enable the identification of complex relationships between microbial communities and host physiology, disease states, or external factors, providing insights that cannot be achieved through single‐omics studies alone. This comprehensive perspective is crucial for advancing personalized medicine, microbiome engineering, and understanding the role of microbes in health and disease.

#### 4.1.4 Metagenomic Sequencing Data

Metagenomic sequencing data were used in 5 of the included studies. Metagenomic sequencing involves extracting and sequencing DNA from a mixed microbial community. Metagenomics approaches sequence all the DNA present in a sample, capturing the genetic information of all organisms in the community, including bacteria, viruses, archaea, fungi, and even host DNA (Thomas et al. 2012). While metagenomic sequencing data is used to acquire microbial abundances (and relative abundances for that matter), we discuss it here as a distinct category to highlight its unique capacity to provide rich functional and genomic information not accessible through targeted amplicon sequencing. In the studies we reviewed, this approach was used to derive data on contiguous DNA sequences (Balaji et al. 2022; Xue et al. 2022; Pan et al. 2022) and evolutionary relationships (Zeng et al. 2022).

### 4.2 Structuring Microbial Data Into Networks

Although microbial network representation methods (Table S1) are primarily focused on forming microbial networks (e.g., for visualization purposes), all the three categories of methods discussed in this review require forming networks from microbial data. As a result, here we describe the different approaches for representing microbial data as a network across all three categories of methods.

When analyzing graphical frameworks employed in microbiome research, we observed a wide range of strategies tailored to specific tasks and objectives. These methods utilize diverse ways of representing data in a network that vary in their topology, uncertainty, scope, temporal integration, and tool availability. Below, we will go over brief topological and functional overviews of the most commonly found connectivity modeling methods throughout our search.

As can be seen in Figure 3A,B, the most common node and edge type, when representing microbial data in a network, are microbes and correlation, respectively. Biologically, the prevalence of microbes as nodes and correlation as edges reflects the field's primary aim of inferring ecological co‐occurrence relationships among taxa, whereas the shift toward similarity‐based edges in the graph‐representation‐learning category reflects a different objective: comparing patient‐level community profiles for prediction rather than inferring direct taxon–taxon interactions. A more nuanced picture emerges when we stratify by our study categories. While microbes remain the most common node type across all categories, the preferred edge type shifts based on the analytical goal. For Category 3 studies, the dominant edge type is similarity, used to compare entities like patient samples rather than inferring interaction between taxa. In fact, for this category, correlation is not among the top three most common edge types, highlighting a distinct focus on other relationships. In contrast, in Category 2 studies, a clear preference emerges for modeling relationships, with correlation and similarity emerging as the most common edge types. This divergence in edge preference underscores how the fundamental research question, whether it be network inference, classical comparison, or predictive modeling, shapes the very structure of the graphs being analyzed.

**FIGURE 3:**
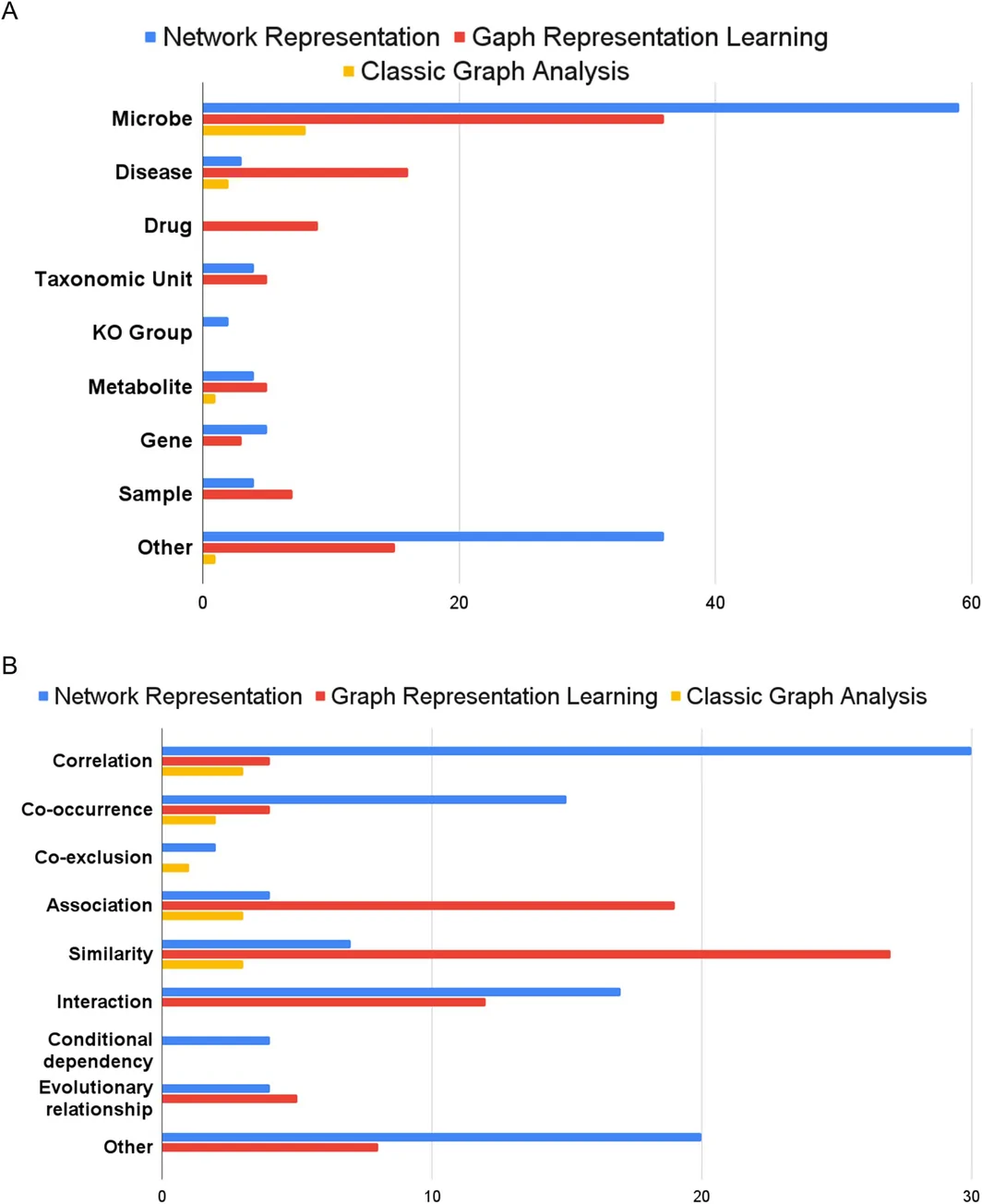
The frequency of node and edge types in the reviewed studies. (A) Bar chart illustrating the frequency of each node type. Here, “KO group” refers to KEGG ortholog group and “Other” refers to the collection of all less common node types across the three types of reviewed studies. (B) Bar chart illustrating the frequency of each edge type. Here, “Other” refers to the collection of all less common edge types across the three types of reviewed studies. The two “Other” categories are panel‐specific: In (A) it denotes less common node types and in (B) less common edge types.

### 4.3 Topological Differences in the Most Common Networks

Below, we discuss the most prevalent graph topologies identified in our survey. These topologies reflect how nodes and edges are structured and are often chosen based on specific research goals and data characteristics. This subsection does not reflect on the types of nodes or edges, only how they are structured. Our review found that the vast majority of studies employed simple graph topologies, represented in 72.26% (*n* = 99) of all included studies. More complex structures like *k*‐partite graphs (*n* = 26, 18.98%) and trees (*n* = 10, 7.3%) were used for more specific tasks, such as modeling multi‐modal interactions or phylogenetic relationships, respectively.

#### 4.3.1 Simple Graphs

Simple graphs are undirected pairs of a set of vertices and a set of edges, characterized as *G* = (*V*, *E*) where *G* is the graph, *V* is the set of its vertices and *E* is the set of its edges. In microbiome research, a simple graph can represent basic associations, where nodes represent an entity (microbes, samples, etc.) and edges denote a single type of relationship (correlation, similarity, interaction, etc.). When the number of edges tends toward zero, a simple graph can be called a sparse graph. Inversely, when the number of edges tends toward the maximum possible number of edges, a simple graph can be called a dense graph. A dense graph where the number of edges equals the maximum number of possible undirected edges is called a complete graph (Gross and Tucker 2001), meaning every distinct pair of nodes are connected by a unique edge, resulting in a maximally connected structure.

#### 4.3.2 *K*‐Partite Graphs

This type of graph is a generalization of the bipartite graph, where the vertices are divided into *k* disjoint sets, and edges only connect vertices from different sets (Gross and Tucker 2001). In microbiome research, the power of *k*‐partite graphs lies in their ability to explicitly model relationships between heterogeneous entities, making them ideal for integrating multi‐modal and multi‐omics data. For example, one set of nodes may represent microbial taxa, another may represent host metabolites, and a third may represent clinical phenotypes. The edges in such a graph do not represent simple co‐occurrence but rather the specific interactions between these different data types. Common applications include modeling microbe‐disease (Lei and Wang 2020; Shi et al. 2023; Liu et al. 2018; Wang, Lei, and Pan 2021; Long et al. 2021), microbe‐drug (Deng et al. 2022; Long, Wu, Liu, et al. 2020; Tan et al. 2022), taxa‐genes (Tian et al. 2020), bacteria‐stage (Hajiramezanali et al. 2020), and even microbe‐disease‐drug (Labus et al. 2019; Chen and Lei 2022; Wang, Lei, and Pan 2021). Pushed even further, *k*‐partite graphs can be used to illustrate molecular interactions across heterogeneous microbial views such as how they are defined by Hasanzadeh, et al. in MoReL (Hasanzadeh et al. 2022).

#### 4.3.3 Trees

Trees are connected, acyclic undirected graphs (Gross and Tucker 2001). In microbiome research, it is commonly used to represent hierarchical relationships among entities (microbial taxonomic units), which generally means that these trees are rooted. The most common application of a tree structure in microbiome studies is the phylogenetic tree, a branching diagram that represents the evolutionary relationships among a set of organisms, typically microorganisms such as bacteria, archaea, or fungi (Richards et al. 2014; Ito et al. 2021; Shtossel et al. 2023; Khan and Kelly 2019). The tree is constructed based on genetic, genomic, or phenotypic similarities and differences, often derived from sequencing data (Khalafvand 2015). FastTree and FastTree2 (Price et al. 2009, 2010) are widely used tools that produce implementations closely aligned with the foundational definition of a phylogenetic tree given above.

### 4.4 Deterministic Versus Probabilistic Graphs

Having explored the different types of graphs commonly used in this field in the previous subsection, we now shift our focus to how their topological features can be represented and analyzed. Specifically, we compare two approaches that offer distinct ways of capturing connectivity patterns within a network: deterministic and probabilistic network structuring. Deterministic representations treat the existence of edges as a matter of certainty, providing a clear‐cut structure for analysis (Heermann 1986). In contrast, probabilistic representations acknowledge that real‐world relationships often involve varying degrees of uncertainty, which makes it important to incorporate this uncertainty into the model itself (Khan et al. 2018). By contrasting these two approaches, we gain insight into when and why one might prefer a strict, fixed framework versus a more flexible, probability‐based one, further enhancing our ability to describe and investigate complex graph structures. Our analysis revealed an overwhelming reliance on deterministic graph structures (*n* = 131, 95.62%).

#### 4.4.1 Deterministic Graphs

As can be seen in Tables [Link], [Link], deterministic graphs represent 95.62% of the type of network representation used throughout all included studies. In deterministic network representations, edge formation, and consequently the relationships they represent, is governed by predefined and immutable criteria. The absence of stochasticity in edge construction ensures that once the defining criteria (e.g., derived from empirical data or established via strict thresholding) are specified, the resulting graph structure is invariant and replicable. Consequently, repeated generation of the representation under identical conditions yields an isomorphic adjacency matrix. This deterministic nature implies that graph connectivity is fully determined and subject to change only through modification of the underlying data or defining rules. In microbiome research, deterministic graphs are typically used to represent a certain association between different entities, such as an interaction score between microbes (Peschel et al. 2021; Bucci et al. 2016; Gibson and Gerber 2018; May et al. 2016; Reiman et al. 2021; Tavakoli and Yooseph 2019; Yooseph and Tavakoli 2022; Lima‐Mendez et al. 2015; Gadhia et al. 2023; Steinway et al. 2015; Laccourreye et al. 2022; Larsen and Claassen 2018; Champion et al. 2024; Kurtz et al. 2015) or a similarity score between samples (Jiang et al. 2015; Zhao et al. 2021; Imangaliyev et al. 2015; Zhang et al. 2015; Narayana et al. 2021; Long and Luo 2019). This inherent rigidity offers advantages in analyses that require precise structural characterization, obviating the need to account for probabilistic fluctuations.

#### 4.4.2 Probabilistic Graphs

As can be seen in Tables [Link], [Link], probabilistic graphs represent only 4.38% (*n* = 6) of the type of network representations used throughout all included studies. Unlike deterministic graphs where edges and vertices are deterministic, probabilistic graphs, also known as uncertain graphs, introduce the concept of existential uncertainty to their edges (Khan et al. 2018; Erdös 1959). This means that each edge is assigned a probability of existence, representing the likelihood that the relationship or connection it signifies is present. Formally, an uncertain graph is often defined as a triplet *G* = (*V*, *E*, *p*), where *V* is the set of vertices, *E* is the set of edges, and *p* is a function that maps each edge in *E* to a probability value between 0 and 1 (Khan et al. 2018). The uncertainty in edges can arise from various sources, including measurement errors, data integration processes, prediction models, or even intentional introduction for privacy purposes. In microbiome research, probabilistic graphs can be used to represent the microbial network along with a posterior probability distribution over all possible graph structures, given the observed data. For example, BDgraph is a Bayesian Gaussian Graphical Model (GGM) that not only models the conditional dependencies between two nodes, but also the probability of the existence of the corresponding edge from the observed data (Vinciotti et al. 2022). In addition, a model such as COZINE represents a microbial graph with stability scores as edges, but also includes an edge based on statistical dependencies. The existence of an edge between any two microbes is not a given, but a conclusion drawn from a penalized regression procedure. Specifically, the model's decision to connect two microbes depends on whether the group‐lasso penalty forces their four interaction parameters (from the multivariate Hurdle model) to be exactly zero (Ha et al. 2020). This decision is sensitive to variations in the input data. To measure this sensitivity, the method employs bootstrapping, where the entire penalized inference process is repeated hundreds of times on datasets created by resampling the original samples. An edge's final stability score, which is a measure of uncertainty, is the frequency with which it was detected across all these bootstrap runs, directly reflecting the statistical confidence in that connection surviving the variability of the data (Ha et al. 2020).

An emerging area within microbiome research involves methods that incorporate time‐series data (Coenen et al. 2020), as it allows for the modeling of temporal dynamics within microbial communities. The most straightforward way of performing longitudinal studies with network representations is using dynamic graphs, where each time point in the dataset has a graph representation. Within the reviewed methods, the Dynamic Bayesian Networks (DBNs) were the most common application of a dynamic graph (with *n* = 3 occurrences (see Table S1) (Laccourreye et al. 2022; McGeachie et al. 2016)). DBNs are a variant of BNs, which extend BNs to modeling temporal dependencies by having each node denoting an entity at a specific time point and edges denoting dependencies across time (Ghahramani 1997). It should be noted that DBNs are not typical dynamic graphs, as they have edges that connect nodes between time slices (inter‐time edges). However, there are methods that incorporate time‐series data to capture microbial dynamics without having a distinct representation for each time point, like the MDSINE (Bucci et al. 2016) and MDSINE2 (Gibson et al. 2021), which aim to capture global microbial dynamics within a single graph representation.

### 4.5 Performing Analysis on Microbial Networks

To complement the descriptive synthesis above, Table 1 compares the major families of graph‐based methods used in microbiome research along their core assumptions, strengths, limitations, interpretability, and typical applications.

**TABLE 1:** Comparison of major graph‐based method families in microbiome research with columns reporting on the Method Family, Core ideas, their Strength, their Limitations, their level of Interpretability, their Typical microbiome tasks, and their respective Reference.

Method family	Core idea/key assumption	Strengths	Limitations	Interpretability	Typical microbiome tasks	References
Correlation/association network inference (SparCC, CCLasso, SPRING, SPIEC‐EASI)	Edges from pairwise statistical association; co‐occurrence used as a proxy for ecological interaction	Simple, fast, well established; human‐readable networks	Correlation is not causation; sensitive to normalization and thresholds; spurious edges from compositionality; static	High	Network construction; keystone/hub identification; co‐occurrence analysis	(Fang et al. 2015; Friedman and Alm 2012; Kurtz et al. 2015; Yoon et al. 2019)
Probabilistic graphical/Bayesian models (BDgraph, COZINE, signed BNs, DBNs)	Model conditional dependencies and edge‐existence probabilities	Quantify uncertainty; handle zero‐inflation; can encode directionality and time	Computationally heavy; limited scalability; prior‐ and assumption‐dependent; small‐sample sensitivity	Moderate–High	Uncertainty‐aware, directional, or temporal network inference	(Vinciotti et al. 2022; Ha et al. 2020; McGeachie et al. 2016; Sazal et al. 2020)
Classical graph analysis (centrality, community detection; random walk, Katz, TDA, SBM, RMT, MCL, WGCNA)	Apply graph‐theoretic metrics to a constructed network	Interpretable, theoretically grounded, low compute; ideal for exploratory work	Descriptive, not predictive; quality bounded by input network; weak on high‐dimensional nonlinear features	High	Keystone species; community/module detection; topology; association prediction	(Zou et al. 2017; Chen et al. 2017; Lymberopoulos et al. 2022; Lutz et al. 2025; Ling et al. 2016; Copeland et al. 2015; Vernocchi et al. 2020; Hall et al. 2019)
Graph neural networks (message passing)	Iterative neighbor aggregation; assumes local homophily/smoothness	Jointly learn topology and features; flexible	Over‐smoothing with depth; need labeled data; data‐hungry; opaque	Low	Microbe‐microbe/‐disease/‐drug link prediction	(Hajiramezanali et al. 2020; Jiang et al. 2022; Chen and Lei 2022; Hasanzadeh et al. 2022)
Graph convolutional networks (GCN)	Spectral/spatial convolution via a normalized adjacency	Strong on graph‐ and node‐classification; integrate multi‐omics	Assume a fixed graph; over‐smoothing; often transductive; opaque	Low–Moderate	Disease (graph) and biomarker (node) classification; link prediction; clustering	(Xu et al. 2022; Lu et al. 2023; Gautam et al. 2022; Gong et al. 2022; Liao et al. 2023; Wang, Shao, et al. 2021)
Graph attention networks (GAT)	Attention‐weighted neighbor aggregation	Learn edge importance; handle heterogeneous neighborhoods; attention aids interpretation	More parameters; still largely opaque; data‐hungry	Moderate	Link prediction; graph classification	(Gautam et al. 2022; Feng, Wu, et al. 2022; Long, Wu, Liu, et al. 2020; Long, Wu, Kwoh, et al. 2020)
Graph autoencoders (GAE/VGAE)	Encoder‐decoder graph reconstruction; VGAE adds a probabilistic latent space	Unsupervised; VGAE models uncertainty; suited to link prediction and incomplete data	Reconstruction objective may miss task signal; latent space not directly interpretable	Moderate	Microbe‐disease/‐drug link prediction; embedding	(Deng et al. 2022; Zhu et al. 2023)
Graph kernels	Similarity via structural substructures; kernel methods	No deep training; effective on small data; dynamic variants for time series	Handcrafted/fixed features; scalability; less expressive than deep models	Moderate	Graph classification; dynamic embeddings for time series	(Melnyk et al. 2020; Pust and Tümmler 2022)
Matrix factorization/NMF	Decompose adjacency/feature matrix into latent factors; non‐negativity gives parts‐based factors	Interpretable factors (NMF); efficient; good for clustering and association prediction	Largely linear; static; struggles with complex nonlinear structure	Moderate–High	Node clustering (modules/guilds); microbe‐disease link prediction	(Cai et al. 2010; Ma et al. 2023; Ma, Zhao, and Ma 2020; Liu et al. 2018)
Random‐walk embeddings (Node2Vec)	Skip‐gram over sampled walks balancing breadth and depth	Scalable; captures structural roles; simple	Vanilla form ignores node features; shallow; static	Low–Moderate	Graph/node classification; link prediction	(Zeng et al. 2022; Lei and Wang 2020)

No single method family is universally optimal; the appropriate choice depends on the research question, the data regime, and the relative priority of prediction versus interpretation. For exploratory questions, such as identifying keystone taxa, delineating co‐occurrence modules, or characterizing community topology, correlation‐based inference and classical graph analysis remain the most practical and interpretable options, particularly for small or compositionally complex datasets. When uncertainty quantification or directionality is required, probabilistic graphical models are preferable despite their greater computational cost. For predictive tasks on larger, feature‐rich datasets, such as disease classification, biomarker identification, and microbe–disease or microbe–drug link prediction, graph representation learning (graph convolutional networks, graph attention networks, and graph autoencoders) generally offers superior performance at the cost of interpretability and greater data and compute requirements.

We note that a direct, quantitative head‐to‐head comparison across the reviewed corpus is not currently feasible, because studies are evaluated on different datasets, tasks, and metrics and the field lacks shared community benchmarks; establishing such benchmarks is itself an important direction for future work. Method selection should therefore be driven explicitly by these trade‐offs rather than by methodological novelty alone.

### 4.6 Analyzing Microbial Graphs With Classical Methods

In this section, we focus exclusively on studies where the primary goal is to study structure, function, and dynamics of microbial communities using “classical” graph analysis methods. These methods are not pure graph representation methods and also are not GRL methods. Such methods help to identify key microbial taxa, uncover community interactions, and detect patterns such as clusters, modularity, and hierarchical organization. A summary of current classical methods for analyzing microbial graphs is shown in Table S2.

Classical graph analysis methods have played a pivotal role in unraveling the complexities of microbiome networks by offering diverse approaches to study their structure and dynamics. Techniques such as Random Walk (Zou et al. 2017) and the Katz Measure (or Katz centrality) (Katz 1953) provide insights into node centrality and similarity by exploring the pathways within microbial interaction graphs (Chen et al. 2017). Topological Data Analysis (TDA) (Lymberopoulos et al. 2022) enables the characterization of global and local patterns, revealing topological features like loops and voids that are crucial in microbiome networks. Probabilistic approaches, including the Bayesian Stochastic Block Model (BSBM) (Lutz et al. 2025) and the Weighted Stochastic Block Model (WSBM) (Hall et al. 2019), help uncover latent community structures and interaction probabilities within weighted networks. Statistical frameworks such as Random Matrix Theory (RMT) (Ling et al. 2016) examine the eigenvalue distributions of microbial correlation matrices to infer systemic properties and detect meaningful interactions. Clustering techniques like Markov Clustering (MCL) (Copeland et al. 2015) identify densely connected subgraphs, aiding in the functional grouping of microbial communities. Lastly, methods like Weighted Gene Co‐Expression Network Analysis (WGCNA) (Vernocchi et al. 2020) leverage co‐expression patterns to infer functional modules in microbiome datasets. Together, these methodologies offer a rich toolkit for analyzing the intricate relationships and functional organization within microbiomes.

### 4.7 Analyzing Microbial Graphs by Learning Embeddings

In some studies, the primary contribution involves the development or application of GRL techniques in microbiome research (*n* = 49). These studies seek to encode microbial data into low‐dimensional vector representations (embeddings) corresponding to nodes, edges, or even the entire graph, enabling powerful downstream machine learning applications. Unlike network analysis methods seen in the previous section, which primarily aim to visualize and analyze the graph's structure, GRL seeks to capture underlying relationships for tasks like node classification and link prediction. GRL leverages deep learning techniques to achieve this, offering a more informative and compact representation compared to classical approaches. By narrowing the scope of this section to GRL, we aim to offer a clear perspective on how these approaches are formulated and applied to advance understanding in the microbiome field.

When analyzing graph representation methods employed in microbiome research, we observed a wide range of strategies tailored to specific tasks and objectives. These methods utilize diverse GRL techniques and vary in their scope, temporal integration, and tool availability. Below, we will go over brief definitions of the most commonly found graph representation methods throughout our search (distribution can be seen in Figure 4A). A summary of current GRL methods for analyzing microbial networks is shown in Table S2.

**FIGURE 4:**
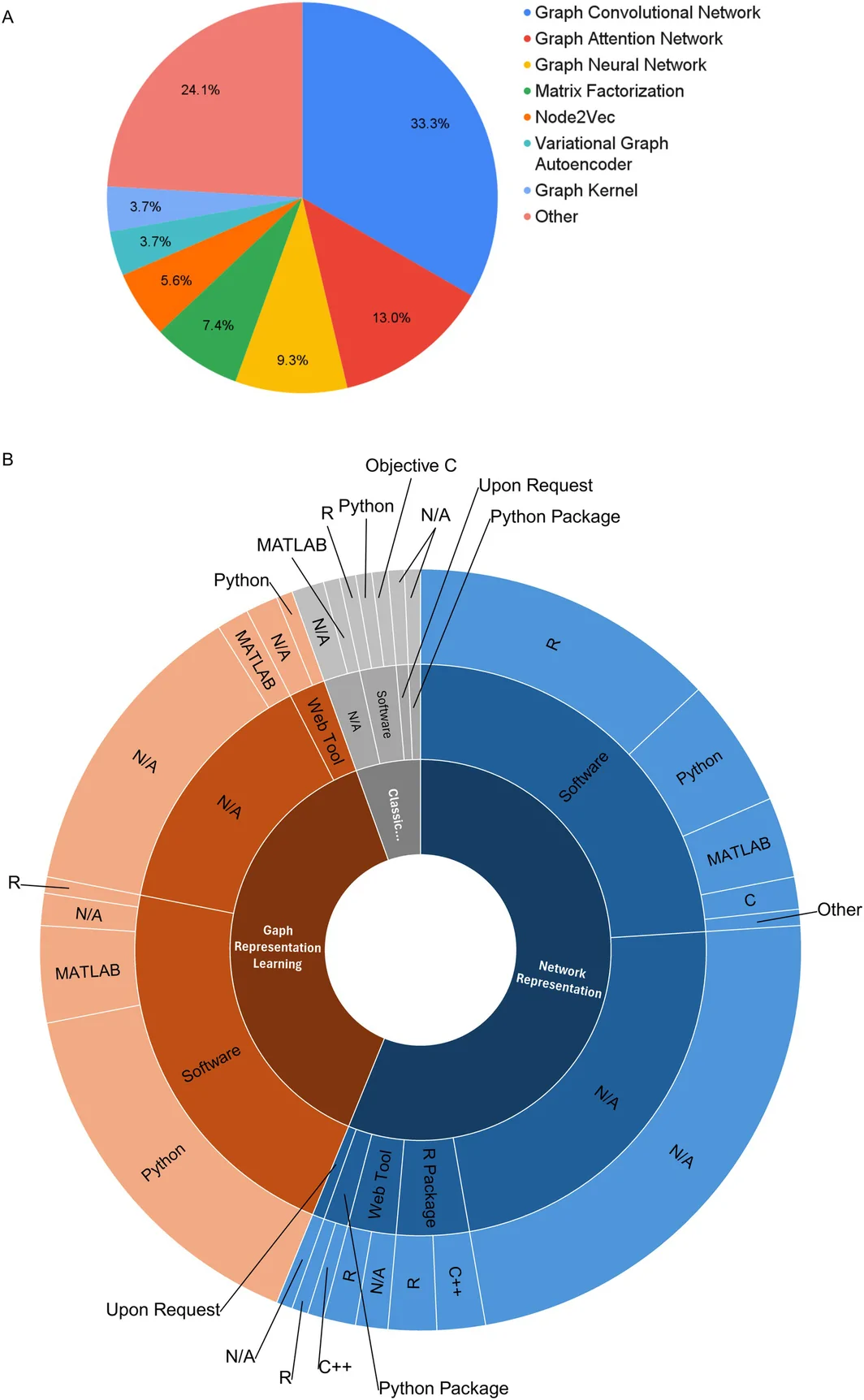
Distribution of methods that rely on graph representation learning and distribution of tool availability. (A) Pie chart illustrating the different graph representation learning methods and their percentage share of studies. Here, “Graph Neural Network” refers to a basic message passing neural network, not the class of graph neural networks found within the reviewed studies. (B) Radial chart illustrating the tool availability and used programming languages. In this figure, “N/A” means “not available” and “Other” combines all remaining, less common languages. The most inner layer corresponds to the different categories of review studies. The second layer represents the manner in which the tools are available. The most outer layer corresponds to the different programming languages which are used to create the tools. The two “Other” categories are panel‐specific: In (A) it denotes less common GRL methods and in (B) less common programming languages.

#### 4.7.1 Graph Neural Networks (GNNs)

Graph Neural Networks (GNNs) represent a powerful class of models for learning from graph‐structured data. However, to distinguish simple GNNs from more complex and specialized architectures, we reserve the term GNN to only refer to the subset of this class that uses simple message passing on a graph. The core mechanism of such GNNs involves nodes iteratively updating their features by aggregating information from their neighbors. This allows the model to learn representations that encode both node attributes and the local graph topology. In our corpus, GNNs were primarily employed for predictive modeling. Notably, within our corpus of retained studies, the application of GNNs was exclusively focused on the task of link prediction (Hajiramezanali et al. 2020; Jiang et al. 2022; Chen and Lei 2022; Hasanzadeh et al. 2022). In the context of microbiome research, this task is used to hypothesize novel or unobserved interactions between microorganisms.

#### 4.7.2 Graph Convolutional Networks (GCNs)

GCNs generalize the concept of convolution from grid‐based data to graphs by aggregating and transforming information from a node's neighbors to update its representation (Kipf 2016). At each layer, a GCN computes a node's new representation as a weighted combination of its own features and the features of its neighbors, scaled by the graph's adjacency matrix (often normalized). This enables GCNs to capture the structural and relational information of graphs. More formally, a GCN layer is defined as:

$$H^{\left(l+1\right)}=\sigma \left({\hat{D}}^{-\frac{1}{2}}\hat{A}{\hat{D}}^{-\frac{1}{2}}H^{\left(l\right)}W^{\left(l\right)}+b^{\left(l\right)}\right)$$

$$\hat{A}=A+I_{n}$$

where A is the adjacency matrix of the given graph, $I_{n}$ is the identity matrix, $\hat{D}$ is the diagonal node degree matrix of $\hat{A}$. $H^{\left(l\right)}$ is the output of the l‐th hidden layer ($H^{\left(0\right)}$ is the node feature matrix). $W^{\left(l\right)}$ is the weight matrix of the hidden layers, and $b^{\left(l\right)}$ is a learnable bias vector for layer l. In each convolutional layer, the model aggregates features from a node's immediate neighbors (1‐hop neighborhood) to compute updated node representations. In the studies included in this review, GCNs were a versatile tool applied to several key predictive tasks. The most common application was graph classification, where the entire microbial network of a sample was used to predict a host‐level phenotype, such as distinguishing between healthy controls and patients with a specific disease (Xu et al. 2022; Lu et al. 2023; Gautam et al. 2022; Gong et al. 2022; Shtossel et al. 2023; Khan and Kelly 2019; Burkholz et al. 2021; Pan et al. 2024). Other studies employed GCNs for node classification, aiming to predict the functional roles of individual taxa or identify them as potential biomarkers based on their connectivity patterns within the community (Liao et al. 2023; Wang, Shao, et al. 2021); link prediction (Lu et al. 2023; Cheng et al. 2022; Shi et al. 2023, 2022; Pi et al. 2022; Yang et al. 2022; Wang, Lei, and Pan 2021); node clustering, where embeddings are used to group microbes into functionally related modules or ecological guilds without pre‐existing labels (Xue et al. 2022); and edge classification, where we categorize the nature of known microbial relationships (Shi et al. 2022; Tian et al. 2023).

#### 4.7.3 Graph Attention Networks (GATs)

GATs are an extension to GCNs by introducing the attention mechanism to the propagation step (Veličković et al. 2017; Vaswani et al. 2017). Unlike GCNs, which use fixed weights based on the graph structure, GATs assign learnable, dynamic attention weights to edges, allowing the network to prioritize more important connections. At each layer, GATs compute node representations by aggregating neighbor features, weighted by their attention scores, which are learned through a self‐attention mechanism. Formally, the single‐head attention mechanism is defined as:

$$H=\text{Attention}\left(Q,K,V\right)=\text{Softmax}\left[\frac{QK^{T}}{\sqrt{d}}\right]V$$

where Q, K, V ∈ *ℝ* ^*N×d*^ represent the query, key and value of this “retrieval” system and *d* is the embedding dimension. In the studies included in this review, GATs were used to get link predictions (Feng, Wu, et al. 2022; Long, Wu, Liu, et al. 2020; Long, Wu, Kwoh, et al. 2020; Liu, Liu, et al. 2021) and graph classification (Gautam et al. 2022).

#### 4.7.4 Graph Autoencoders (GAEs)

Autoencoders (AEs) are a type of encoder‐decoder neural network that is trained in an unsupervised manner to copy its input to its output by learning a compressed representation (embedding) of the input data in order to ignore noise and focus on certain patterns. GAEs extend the AE architecture onto graph data by applying a GCN as an encoder, mapping nodes or graph features into a latent feature space, and a decoder to reconstruct the graph. An extension to GAEs, the Variational GAE (VGAE), looks to train the GAE in a variational way by introducing a probabilistic framework. Instead of encoding nodes into fixed latent vectors, VGAEs encode them as distributions (e.g., Gaussian distributions) with learnable means and variances. This enables VGAEs to model uncertainty in the latent space and generate new graph structures by sampling from these distributions (Kipf and Welling 2016), making them particularly suitable for applications like graph generation, unsupervised learning, and handling incomplete data. In the reviewed studies, VGAEs were used for link prediction (Deng et al. 2022; Zhu et al. 2023).

#### 4.7.5 Graph Kernels

Graph kernels are similarity measures designed to compare graph‐structured data by capturing and quantifying their structural and attribute‐based similarities. They work by mapping graphs into a high‐dimensional feature space, where traditional machine learning algorithms can be applied. Graph kernels operate on various graph properties, such as node degrees, substructures (e.g., paths, cycles, or subtrees), or spectral characteristics (Vishwanathan et al. 2010). Unlike traditional methods that rely on explicit graph representations, graph kernels offer a more abstract and efficient means of capturing the inherent complexities within interconnected data. In the reviewed studies, graph kernels were used to classify graphs (Pust and Tümmler 2022) or learn dynamic graph embeddings for time‐series analysis (Melnyk et al. 2020).

#### 4.7.6 Matrix Factorization

Matrix factorization is a mathematical technique used to decompose a matrix into the product of two or more smaller matrices, typically to reveal underlying patterns or structure in the data. It is widely used in dimensionality reduction, recommendation systems, and data compression (Koren et al. 2009). Common methods include Singular Value Decomposition (SVD), Non‐negative Matrix Factorization (NMF), and Principal Component Analysis (PCA). Because NMF was the most common method in the reviewed articles, we will provide a more detailed definition of it: NMF is a specific type of matrix factorization that decomposes a non‐negative matrix *V* into two smaller non‐negative matrices *W* and *H*, such that *V*≈*WH*. Here, *W* captures the latent features, and *H* represents the weights or contributions of these features. The non‐negativity constraint ensures that the components are interpretable, making NMF particularly useful for tasks like topic modeling, image analysis, and bioinformatics. In the microbiome studies we analyzed, matrix factorization techniques were leveraged for two principal tasks. The most common application was node clustering (Cai et al. 2010; Ma et al. 2023; Ma, Zhao, and Ma 2020). In this approach, the adjacency matrix of a microbial network is decomposed, and the resulting low‐dimensional representations (often from the *H* matrix) are used to group microbes into functionally related modules or ecological guilds. This allows for the data‐driven discovery of hidden community structures without relying on pre‐existing labels. The second major use was for link prediction (Liu et al. 2018).

#### 4.7.7 Node2Vec

Node2Vec is a machine learning algorithm for generating node embeddings in a graph by capturing both local and global structural information. It combines random walks with a biased sampling strategy to explore neighborhoods of nodes, balancing breadth‐first (BFS) and depth‐first (DFS) search (Grover and Leskovec 2016). The resulting node sequences are treated like sentences in natural language processing, and embeddings are learned using techniques like Word2Vec. These embeddings encode structural and relational information about the graph, which are used for graph classification (Zeng et al. 2022) or link prediction (Zeng et al. 2022; Lei and Wang 2020).

GRL models have emerged as powerful tools in microbiome research, offering novel ways to analyze and interpret complex microbial ecosystems and their broader associations. Link prediction is widely used to infer missing or potential interactions within microbial networks, such as identifying unobserved symbiotic relationships or antagonistic interactions between taxa. Beyond microbial networks, it is also employed to predict associations between microbes and drugs (Deng et al. 2022; Long, Wu, Kwoh, et al. 2020; Tan et al. 2022; Pi et al. 2022; Tian et al. 2023), microbes and diseases (Lu et al. 2023; Cheng et al. 2022; Gong et al. 2022; Lei and Wang 2020; Wang et al. 2017; Shi et al. 2023, 2022; Liu, Liu, et al. 2021; Zhu et al. 2023; Yin et al. 2020; Liu et al. 2018), or drugs and diseases and microbes (Long, Wu, Liu, et al. 2020; Chen and Lei 2022; Wang, Lei, and Pan 2021). Graph classification models categorize graph representations based on their overall structure, facilitating tasks like distinguishing microbiomes associated with health versus disease. For example, DeepToA (Zeng et al. 2022) used Node2Vec for predicting a microbiome sample's theater of activity (ToA) based on its taxonomic and functional profiles. Node classification assigns labels to individual nodes to predict their functional roles, such as whether a bacterium is pathogenic or beneficial, or whether a patient's bacterial composition is healthy or diseased. This could be seen in GDmicro (Liao et al. 2023), where the model compared similarity scores between patient samples to predict their health state (healthy vs. disease). Finally, node clustering groups nodes with similar properties or behaviors, uncovering microbial communities or co‐occurrence patterns that reveal ecological niches or functional modules within the microbiome. RepBin (Xue et al. 2022) demonstrates this use‐case by separating different contiguous DNA sequences into “bins” of similar nodes in the graph. This allowed them to identify contigs that are most likely derived from the same microbial genome. These GRL tasks provide powerful methods for advancing our understanding of microbial dynamics, interactions, and their implications for human health.

A growing subset of methods operates on heterogeneous and knowledge‐graph representations that integrate multiple node and edge types (e.g., microbes, diseases, drugs, metabolites). Examples in our corpus include knowledge‐graph neural networks such as KGNMDA (Jiang et al. 2022), metapath‐based heterogeneous GNNs (Chen and Lei 2022), and heterogeneous‐information‐network models for phage–bacteria and microbe–disease prediction (Long and Luo 2019; Pan et al. 2023). These approaches are particularly suited to multi‐omics and multi‐entity integration, and we expect their role to expand most substantially where multi‐entity structure confers capabilities that association‐only methods lack. Because these representations make intermediary entities (metabolites, genes, pathways) explicit, the types of paths they encode can serve as candidate mechanistic hypotheses rather than opaque scores which moves predictions toward mechanism (Chen and Lei 2022).

Just as there is a growing need for integrating time‐series data into graph representation methods in microbiome research, it is equally crucial to incorporate a temporal component into GRL techniques. Traditional GRL methods often focus on static graphs, which can oversimplify these systems and miss important temporal dependencies. Incorporating a temporal dimension can enable GRL methods to capture not just the state of a graph at a single moment but also the progression and evolution of its structure and features over time. Popular sequential models for time‐series analysis in deep learning, such as Long Short‐Term Memory (LSTM) networks and Transformers, have shown significant promise in capturing temporal dependencies and complex sequential patterns (Lim and Zohren 2021). Adapting these models for GRL offers a pathway to enhance the ability of GRL techniques to process dynamic graphs and better reflect the underlying temporal processes. Despite their potential, the integration of temporal components into GRL remains underexplored. Only 3 of the reviewed studies have explicitly incorporated time as a studied variable (see Table S3), highlighting a significant gap in the literature.

## 5 Availability of Tools

### 5.1 Network Construction and Visualization Tools

Network representation is extensively used in microbiome research to model and represent complex systems, from microbial interaction networks based on co‐occurrence or co‐abundance patterns to patient‐level bacterial community profiles. They are the bulk of search results when looking for graph‐based analysis methods. These representations allow researchers to uncover microbial community structure, identify key taxa or interactions associated with specific conditions, and integrate multi‐omics data for a holistic understanding of microbial ecosystems. Therefore, making these methods and tools readily available and user‐friendly is crucial for empowering researchers to explore microbial ecosystems, advance our understanding of microbiome diseases, and translate findings into actionable insights for health management.

In spite of this important requirement, among the 80 Network Representation method articles reviewed, 37 (~46%) of the methods were not publicly available at the time of the study, highlighting a significant gap in accessibility and reproducibility within the field (Figure 4B). Of the tools that were accessible, R emerged as the most widely used programming language for implementing graph‐based methods, reflecting its popularity in statistical and bioinformatics research. In contrast, Python, while increasingly favored for its versatility and robust ecosystem of libraries, was a distant second in terms of adoption, underscoring a potential opportunity for further development and standardization of Python‐based tools in microbiome research; see Figure 4B.

### 5.2 Classical Graph Analysis Tools

Classical network analysis methods remain desirable compared to more deep‐learning‐based methods due to their interpretability, robustness, and established theoretical foundations. These methods, such as centrality measures, community detection, and network motif analysis, offer straightforward insights into the structure and dynamics of networks, making them ideal for exploratory studies and hypothesis generation. Unlike GRL, which often operates as a black box, classical methods provide results that are easier to explain and validate in many scientific and applied contexts. Additionally, they are computationally less demanding and require fewer data preprocessing steps, making them accessible for smaller datasets or resource‐constrained applications. Their effectiveness in many domains, coupled with their ability to highlight specific topological features of networks, ensures their continued relevance in network science. Their effectiveness in highlighting specific topological features of networks ensures their continued relevance, making it important to keep these tools readily available for researchers to use and/or explore. However, out of the 8 methods corresponding to this category in our study, only 3 of them had their code publicly available, with an additional study available only upon request from the authors (Figure 4B).

### 5.3 Graph Representation Learning Tools

This category corresponds to encompass many of the deep learning‐based approaches, which have shown to provide improvements over classical network analysis methods and traditional graph representation techniques. Classical approaches are often limited to predefined heuristics and may struggle to capture dynamic, high‐dimensional, or nonlinear relationships inherent in complex systems. Similarly, pure graph representations are invaluable for visualizing a problem setting and providing an intuitive understanding of the relationships within a system; they often fall short when it comes to analyzing and extracting complex patterns. In contrast, modern deep learning‐based methods allow for end‐to‐end learning that can incorporate both the topology of the graph and node/edge‐level features. These approaches dynamically learn representations tailored to specific tasks, enabling better performance on predictive modeling, clustering, or anomaly detection. This is why it is important to make these findings available to other researchers.

Among the 49 reviewed GRL articles in our study, 19 of them were either unavailable or partially unavailable at the time of research, 3 of them were implemented as usable web tools and an overwhelming majority of them as downloadable software from GitHub (Figure 4B). Of the tools that were accessible, Python emerged as the most widely used programming language for implementing graph‐based methods, reflecting its popularity within the deep learning community (Figure 4B). The higher percentage of code and software availability for this category of methods compared to the other categories is encouraging, yet more improvement is necessary to ensure all such software remains available. The prevalence of R among network‐representation and classical tools versus Python among graph‐representation‐learning tools reflects the disciplinary origins of each approach (statistical ecology versus deep learning) and signals a fragmentation that complicates building unified, reproducible workflows. All three sections are summarized in Table S4.

## 6 Challenges and Limitations

While graph‐based analysis techniques have shown great promise in microbiome research, several challenges and limitations persist.

### 6.1 Microbiome‐Specific Analytical Challenges

Several properties of microbiome data complicate graph construction and graph representation learning in ways that are not fully shared by other application domains. First, sequencing data are compositional: abundances are constrained to a constant sum, which can induce spurious negative correlations and distort naively inferred association networks, motivating compositionally aware inference methods such as SparCC (Friedman and Alm 2012), SPIEC‐EASI (Kurtz et al. 2015), and SPRING (Yoon et al. 2019). Second, the data are highly sparse and zero‐inflated, with a mixture of biological (structural) and technical (sampling) zeros that bias both correlation estimates and learned embeddings, motivating zero‐inflation‐aware models such as COZINE (Ha et al. 2020). Third, microbiome studies are typically high‐dimensional but sample‐limited, with features greatly outnumbering samples, which raises a substantial risk of overfitting for data‐hungry deep graph models. Fourth, technical and batch variation arising from sequencing depth, platform, and bioinformatic pipeline affects the reproducibility of any constructed graph. Fifth, taxonomic resolution and reference‐database dependence, including the choice between operational taxonomic units and amplicon sequence variants, directly alter node definitions and therefore downstream results. Finally, the absence of ground‐truth interaction networks makes validation and benchmarking of inferred graphs intrinsically difficult. These challenges should inform both method choice and the interpretation of all graph‐based results in microbiome research.

### 6.2 Tool Availability and Reproducibility

A significant hurdle in applying graph and network methods to microbiome research is the persistent gap between theoretical advancements and practical implementation. While numerous innovative techniques have been proposed in academic literature, their utility is often restricted by the absence of publicly available, well‐maintained software tools. This gap is particularly evident when exploring the different advanced network analysis methods in microbiome research. As highlighted in Tables [Link], [Link], many theoretically promising methods either lack publicly accessible implementations or are distributed in ways that make them challenging to use, such as poorly documented repositories or outdated codebases (which could be incompatible with modern computational environments). The unavailability of such tools not only impedes reproducibility but also discourages new researchers from entering the field due to the steep learning curve required to reimplement these methods from scratch.

Moreover, this lack of accessible tools hinders collaborative efforts and limits the ability of researchers to perform comparative analyses, which are critical for understanding the relative strengths and weaknesses of different methods. Without widely available and standardized software, benchmarking becomes inconsistent, and the broader community struggles to coalesce around best practices.

### 6.3 Probabilistic Graphs

As can be seen in Tables [Link], [Link], the majority of graphs used in microbiome research are deterministic in nature, with no GRL or classical graph analysis methods applied to any probabilistic graphs. These approaches offer valuable initial insights as they are relatively straightforward to construct and interpret, providing a clear visualization of potential relationships between microbial taxa or functions. They can effectively highlight strong associations and generate hypotheses about community structure. However, deterministic graphs assume fixed relationships and fail to explicitly account for the inherent stochasticity and complexity of microbial ecosystems.

In contrast, a probabilistic graph represents relationships with a degree of uncertainty, where each potential edge in the graph is assigned a probability value (Erdös 1959). In microbial network analysis, this allows researchers to construct a network where each edge might represent the statistical confidence of a co‐occurrence pattern or the modeled probability of a metabolic interaction. A primary advantage of this approach is the ability to filter and analyze the network based on these probabilities. We believe further research in developing novel methods for utilization of probabilistic graphs in microbial networks is needed to fully take advantage of this approach.

### 6.4 Time‐Series Analysis

Another critical limitation in the application of graph and network methods to microbiome research is the challenge of incorporating time‐series data into these analytical frameworks. Time‐series data, which captures the dynamic changes in microbial communities over time, is crucial for understanding the temporal interactions and shifts within the microbiome, especially in response to perturbations such as diet, medication, or disease. However, most existing graph‐based methods are designed around static networks, focusing on relationships at a single time point or averaged across time. This limitation overlooks the inherently dynamic nature of microbial ecosystems, where temporal dependencies and sequential patterns often hold key insights.

While some recent advancements, such as dynamic graph neural networks or temporal network modeling, offer potential solutions, their application in microbiome research remains limited due to the complexity of implementation and the scarcity of time‐resolved datasets. Moreover, integrating temporal data into graph models often requires additional preprocessing steps, such as interpolating missing data points or aligning unevenly spaced time points, which can introduce noise and bias.

### 6.5 Model Interpretability

Interpretability is a cornerstone of microbiome research, as biologists rely on clear, transparent explanations to validate computational predictions and derive actionable insights about microbial ecosystems. This necessity makes the challenge of limited interpretability in GRL models particularly pressing. While some GRL methods, such as variational graph autoencoders (VGAEs), are considered inherently more interpretable due to their generative nature, they often lack explicit, quantifiable mechanisms to explain predictions, leaving biologists without the detailed insights they require.

Currently, most GRL applications in microbiome research prioritize performance metrics, such as accuracy and precision, over interpretability. Explicit interpretability frameworks such as SHAP (SHapley Additive exPlanations) values (Lundberg and Lee 2017), LIME (Local Interpretable Model‐agnostic Explanations) (Ribeiro et al. 2016), or Grad‐CAM (Selvaraju et al. 2017), are rarely integrated into these models. In fact, out of all the reviewed GRL models, only two of them implemented an explicit interpretability framework: DeepToA implements SHAP values (Zeng et al. 2022) and gMic implements Grad‐CAM (Shtossel et al. 2023). Baranwal et al. (2021), a network‐representation method rather than a GRL model, also incorporates LIME scores within its graph representation. As a result, many of the reviewed GRL models function as black boxes, offering little understanding of how features, such as microbial abundances or taxonomic interactions, contribute to predictions. This opacity can impede hypothesis generation, validation, and the ability to translate computational findings into biological or clinical applications.

Beyond model‐agnostic tools, a family of graph‐specific interpretability methods is directly applicable to microbiome graph representation learning but remains underused. Methods such as GNNExplainer (Ying et al. 2019) and PGExplainer (Luo et al. 2020) identify the subgraph and node features most responsible for a prediction; attention weights in graph‐attention models can be inspected to rank influential edges; and integrated‐gradients or subgraph‐ and motif‐attribution approaches can localize predictive structure. The value of these methods lies in translating learned representations into biologically testable hypotheses, for example by mapping an important predictive subnetwork back to specific taxa and their inferred interactions, which can then be prioritized for experimental validation. Embedding such graph‐specific explanations as a standard component of model reporting would substantially improve the biological actionability of these findings.

### 6.6 Biological Validation

A further consideration concerns the extent to which computational predictions are biologically validated. Across the reviewed studies, validation is predominantly computational: cross‐validation and ranking metrics, recovery of curated associations from databases such as HMDAD (Ma et al. 2017), and literature‐based case studies in which top‐ranked predictions are corroborated against previously reported findings. Prospective experimental or wet‐lab validation of novel predicted interactions or biomarkers is comparatively rare. This limits the immediate translational interpretation of many results and represents an important direction for future work, in which computational pipelines are coupled with targeted experimental confirmation.

## 7 Future Directions and Emerging Trends

Addressing the limitations identified in this review will require focused innovation. We see the next wave of progress in graph‐based microbiome research emerging from five key areas, each directly responding to a current challenge, together with several emerging methodological trends that we outline below.

### 7.1 Overcoming Static Snapshots

A critical limitation of current methods is their focus on static networks. The future lies in embracing the temporal nature of microbial ecosystems. This can be achieved through two complementary approaches. The first involves modeling microbial dynamical systems, where the goal is to learn the parameters of the governing differential equations that describe community changes over time, as explored by (Stein et al. 2013) and (Melnyk et al. 2023, 2020). The second involves leveraging temporal graph representations. Methods like Dynamic Bayesian Networks (DBNs), Temporal Event Networks, or temporal GNNs can explicitly model the sequential dependencies between time points, capturing how past community states influence future ones. These dynamic approaches are essential for understanding microbial succession, responses to perturbations, and the trajectory of disease.

Temporal graph learning offers several modeling strategies beyond dynamic Bayesian networks. Discrete‐time approaches assign a graph snapshot to each time point and model their evolution, as in temporal and dynamic graph neural networks, while continuous‐time methods and dynamic embeddings, such as the Koopman‐based approach used by GraphKKE, capture evolution without explicit discretization. Sequence models can also be coupled with graph encoders to capture temporal dependencies in community state. Adoption in microbiome research is constrained by domain‐specific obstacles, including irregular and sparse sampling, short time series, and compositional drift over time, which complicate the construction of well‐aligned dynamic graphs. Addressing these obstacles, alongside the scarcity of densely sampled longitudinal datasets, will be essential for realizing the promise of temporal graph methods.

### 7.2 Moving Beyond Black Boxes

To solve the critical challenge of model interpretability, the integration of Explainable AI (XAI) frameworks must become standard practice, not an afterthought. While our review found only a handful of studies implementing XAI, future work should systematically incorporate methods like SHAP (Lundberg and Lee 2017), LIME (Ribeiro et al. 2016), or Grad‐CAM (Selvaraju et al. 2017). Going further, the development of inherently interpretable or “glass‐box” models, such as those inspired by layer‐wise relevance propagation (LRP) (Montavon et al. 2019), will be crucial. The goal is to create models that not only predict accurately but can also pinpoint the specific taxa, interactions, or sub‐networks driving a given prediction, thereby providing actionable biological hypotheses.

### 7.3 Solving the Software Availability Problem

Finally, to address the challenge of tool availability and reproducibility, the field needs a concerted effort to build standardized and benchmarked software platforms. Future work should focus not just on developing new algorithms, but on implementing them in accessible, well‐documented packages. A community‐driven platform that includes benchmark datasets, standardized APIs for different graph models, and leaderboards for various predictive tasks (e.g., disease classification, link prediction) would dramatically lower the barrier to entry, foster collaboration, and accelerate methodological advancement in the field.

Concretely, accessibility and reproducibility could be improved through mandatory deposition of code and data at submission, persistent archiving with versioned DOIs (e.g., Zenodo), containerization (e.g., Docker/Singularity) and explicit environment specifications to ensure long‐term executability, adherence to the FAIR principles, standardized input/output formats (e.g., BIOM), and provision of web‐server or workflow‐platform implementations to lower the barrier for non‐programmer users.

### 7.4 Modeling Uncertainty With Probabilistic Graphs

A further direction is the wider adoption of probabilistic graph methods that explicitly represent uncertainty in inferred microbial relationships. As noted above, the overwhelming majority of reviewed studies rely on deterministic graphs, yet microbiome data are noisy, sparse, and compositional, conditions under which point‐estimate networks can be misleading. Probabilistic and Bayesian graph models, including approaches that propagate edge‐existence probabilities into downstream prediction, would allow uncertainty to be quantified and communicated, improving the reliability of biological conclusions.

### 7.5 Toward Foundation Models, Graph Transformers, and Multimodal Integration

Finally, several emerging methodological trends are likely to shape the next generation of graph‐based microbiome models. Graph transformers and attention‐based architectures can capture long‐range dependencies that message‐passing networks struggle to model, while self‐supervised pretraining on large unlabeled microbiome corpora points toward microbiome‐specific foundation models that could be fine‐tuned for diverse downstream tasks. In parallel, heterogeneous and knowledge‐graph approaches that integrate multi‐omics, host, and environmental data, coupled with advanced graph‐specific explainability frameworks, offer a path toward models that are both more expressive and more interpretable. Realizing these possibilities will require larger standardized datasets, shared benchmarks, and close collaboration between method developers and microbiome biologists.

## 8 Conclusions

This systematic review has synthesized the rapidly expanding and fragmented landscape of graph‐based methods in microbiome research. By structuring the literature into three core domains (network representation, classical graph analysis, and GRL) we have provided a clear framework for understanding the state of the art. Our analysis revealed a field rich with innovation but also constrained by recurring challenges: a lack of standardized and accessible tools, a reliance on static network models that overlook microbial dynamics, and the critical need for improved interpretability in advanced deep learning models.

The insights and gaps identified here serve as a roadmap for the future. Progress in this domain will depend on addressing these specific limitations, particularly through the development of temporal graph methods, the integration of explainable AI (XAI), and community‐wide efforts to build benchmarked and reproducible software. By bridging the gap between computational power and biological application, the field can move beyond descriptive analysis toward predictive and mechanistic models. Ultimately, a thoughtful and targeted evolution of these graph‐based techniques is essential to unlock their full potential and translate complex microbiome data into actionable insights for human health and disease.

## Author Contributions

**Cedrique Shum‐Tim:** data curation, formal analysis, conceptualization, methodology, validation, writing – review and editing, writing – original draft. **Paromita Islam:** conceptualization, data curation. **Jacqueline L. Boyajian:** visualization, writing – review and editing. **Ahmed Abosalha:** data curation, formal analysis, writing – review and editing. **Amal Kassab:** data curation, investigation, methodology. **Madison Santos:** conceptualization, software. **Kinaan Aamir Khan:** methodology, software. **Kiri Stern:** methodology, software. **Amin Emad:** writing – review and editing, supervision. **Satya Prakash:** conceptualization, writing – review and editing, funding acquisition, software, methodology, supervision.

## Funding

The authors thank the anonymous reviewers for their valuable suggestions. This work is supported by the Canadian Institute of Health Research (CIHR; Grant No. 252743) awarded to Prakash S. Boyajian JL is fully funded by the Fonds de Recherche du Québec—Santé (FRQS; 335999). Islam P is funded by the Islamic Development Bank Scholarship (2020‐245622). Abosalha A is fully funded by a scholarship from the Ministry of Higher Education of the Arab Republic of Egypt. Madison S is funded by the Canadian Graduate Scholarship‐Masters from the Natural Sciences and Engineering Research Council of Canada (NSERC; 504933) and the Fonds de Recherche du Québec—Santé (FRQS; 351337). The funders had no role in study design, data collection and analysis, interpretation of the data or writing of the manuscript.

## Conflicts of Interest

The authors declare no conflicts of interest.

## Supporting information


**Table S1:** Summary of reviewed papers that exclusively propose a novel method for microbial Network Representation, with columns “Name” (the method name), “Graph Topology”, “Graph Type” (uncertainty), “Data Type(s)”, “Node Type”, “Edge Type”, “Task” (the overall goal of the representation), “Task Details” (how the study arrives to the task), “Time‐Series” (whether or not the study used time‐series data), “Scope” (whether the graph represents the dataset on an individual and/or community level), “Dynamic” (whether the graph representations in the form of dynamic graphs), “Availability” (the method's manner of availability), “Data Provided”, “Language”, “Last Updated”, “Preprocessing Scripts”, and “Link”. All “N/A” denote information that is “not available”. The table is provided as a separate xlsx file.

**Table S2:** Summary of reviewed papers that exclusively propose a Classic Graph Analysis method for analyzing microbial graphs, with columns “Name” (the method name), “Model Type”, “Data Type(s)”, “Graph Topology” (of the model input graph), “Graph Type” (uncertainty of the model input graph), “Node Type” (of the model input graph), “Edge Type” (of the model input graph), “Task” (the overall goal of the classic graph analysis method), “Task Details” (how the study arrives to the task), “Time‐Series” (whether or not the study used time‐series data), “Scope” (whether the input graph represents the dataset on an individual and/or community level), “Dynamic” (whether the input graph representations are in the form of dynamic graphs), “Availability” (the method's manner of availability), “Data Provided”, “Language”, “Last Updated”, “Preprocessing Scripts”, and “Link”. All “N/A” denote information that is “not available”. The table is provided as a separate xlsx file.

**Table S3:** Summary of reviewed papers that exclusively propose a Graph Representation Learning method for analyzing microbial graphs, with columns “Name” (the method name), “Model Type”, “Data Type(s)”, “Graph Topology” (of the model input graph), “Graph Type” (uncertainty of the model input graph), “Node Type” (of the model input graph), “Edge Type” (of the model input graph), “Task” (the overall goal of the GRL model), “Task Details” (how the study arrives to the task), “Time‐Series” (whether or not the study used time‐series data), “Scope” (whether the input graph represents the dataset on an individual and/or community level), “Dynamic” (whether the input graph representations are in the form of dynamic graphs), “Availability” (the method's manner of availability), “Data Provided”, “Language”, “Last Updated”, “Preprocessing Scripts”, and “Link”. All “N/A” denote information that is “not available”. The table is provided as a separate xlsx file.

**Table S4:** Summary of software availability across the three methodological categories, aggregated from the studies reviewed here. Counts are derived from the figures reported in the Availability of Tools section. Per‐method details, including repository links, implementation language, the date each tool was last updated (a proxy for maintenance), and whether preprocessing scripts and data were provided (proxies for reproducibility), are recorded for each study in Tables [Link], [Link]; documentation presence, documentation quality, and benchmarking across tools remains an avenue for future work (Marcos‐Zambrano et al. 2023).

## Data Availability

Research data are not shared.
